# A Bioinformatic Workflow for InDel Analysis in the Wheat Multi-Copy α-Gliadin Gene Family Engineered with CRISPR/Cas9

**DOI:** 10.3390/ijms222313076

**Published:** 2021-12-03

**Authors:** María H. Guzmán-López, Miriam Marín-Sanz, Susana Sánchez-León, Francisco Barro

**Affiliations:** Department of Plant Breeding, Institute for Sustainable Agriculture-Spanish National Research Council (IAS-CSIC), 14004 Córdoba, Spain; mhguzman@ias.csic.es (M.H.G.-L.); mmarin@ias.csic.es (M.M.-S.); ssanchez@ias.csic.es (S.S.-L.)

**Keywords:** Usearch, multi-copy, multi-target, multi-gene, InDels, CRISPR/Cas, gluten, wheat, celiac disease, gliadins

## Abstract

The α-gliadins of wheat, along with other gluten components, are responsible for bread viscoelastic properties. However, they are also related to human pathologies as celiac disease or non-celiac wheat sensitivity. CRISPR/Cas was successfully used to knockout α-gliadin genes in bread and durum wheat, therefore, obtaining low gluten wheat lines. Nevertheless, the mutation analysis of these genes is complex as they present multiple and high homology copies arranged in tandem in A, B, and D subgenomes. In this work, we present a bioinformatic pipeline based on NGS amplicon sequencing for the analysis of insertions and deletions (InDels) in α-gliadin genes targeted with two single guides RNA (sgRNA). This approach allows the identification of mutated amplicons and the analysis of InDels through comparison to the most similar wild type parental sequence. TMM normalization was performed for inter-sample comparisons; being able to study the abundance of each InDel throughout generations and observe the effects of the segregation of Cas9 coding sequence in different lines. The usefulness of the workflow is relevant to identify possible genomic rearrangements such as large deletions due to Cas9 cleavage activity. This pipeline enables a fast characterization of mutations in multiple samples for a multi-copy gene family.

## 1. Introduction

The genome editing tools, such as clustered regularly interspaced short palindromic repeats (CRISPR) and its CRISPR-associated protein (Cas), are revolutionizing plant science due to their simplicity and versatility. The type II CRISPR/Cas9 system is a bacteria adaptive immune response system that uses non-coding RNAs to guide the Cas9 nuclease to induce site-specific DNA cleavages [1]. The main feature is its simplicity of use; the system can be engineered as a 20 nucleotides single guide RNA (sgRNA) to target any gene of interest. The only requirement is the existence of a Cas9 recognition sequence known as protospacer adjacent motif (PAM). Then, Cas9 introduces a break in the double-stranded DNA 3-bp upstream of the PAM. This DNA damage is repaired by cellular DNA repair mechanisms, either via the non-homologous end-joining DNA repair pathway (NHEJ) or the homology-directed repair (HDR) pathway. Because NHEJ is an error-prone DNA repair pathway, insertions and deletions (InDels) are introduced which can produce frameshifts and stop codons, leading to functional knockout of the gene [2]. CRISPR/Cas technology provides a rapid way to generate new genetic variability by removing genes responsible for undesired traits or introducing gain-of-function mutations through precise genome editing. Many crops and traits have been targeted by CRISPR/Cas, including yield, quality, disease resistance, and herbicide resistance [3].

Wheat is the most cultivated cereal in the world thanks to the high adaptability, performance, and unique viscoelastic properties of its dough [4]. Gluten proteins are responsible for these properties and represent about 80% of the total grain protein content in this cereal [5]. Gluten is composed of two different protein groups: gliadins and glutenins. The first ones are mainly monomeric and divided into three structural groups: ω-, α/β- and γ-gliadins, while glutenins are polymeric and divided into the High Molecular Weight (HMW) and the Low Molecular Weight (LMW) glutenin subunits [6]. Both gliadins and glutenins establish a complex protein network in the wheat dough which captures the CO_2_ released during fermentation, providing bread wheat with its unique viscoelastic characteristics [5].

However, gluten proteins also activate the immune response in celiac disease (CD) [7,8], a chronic enteropathy related to human leukocyte antigen (HLA) HLA-DQ2 and HLA-DQ8 in predisposed individuals. CD affects a rising 1% of the population in western countries [9,10], which reaches 1.5% in Northern European countries [11]. Although many epitopes in gluten peptides have been described, the gliadins fraction, and particularly the α-gliadins, are the gluten fractions providing the most stimulatory epitopes associated with CD [12,13]. Nowadays, the treatment for CD and other wheat-related pathologies, such as non-celiac wheat sensitivity (NCWS) is to follow a gluten-free diet (GFD) for life. Nonetheless, maintaining a GFD is a demanding task as both gluten and wheat are widely used in the food industry. This leads to transgressions in the diet which can be up to 60% [14].

Hence, the elimination of wheat proteins responsible for these pathologies is an appealing goal as it would allow wheat varieties without immunoreactivity while preserving wheat organoleptic characteristics, which most gluten-free foods lack [15]. RNA of Interference (RNAi) and CRISPR/Cas technologies have been successfully used for the down-regulation and knock-out of gliadin genes in bread and durum wheat, providing wheat lines with low gluten, and low stimulatory capacity of triggering CD and NCWS [5,16,17]. The management of RNAi lines is relatively straightforward as it is a dominant trait, making it easy to screen out wheat lines with the desired genes silenced, and even to transfer this trait to elite varieties as all the recipient genes will be silenced as well. Despite this, RNAi wheat lines are considered genetically modified organisms (GMOs) worldwide while CRISPR-edited lines are not in many countries [18], which would facilitate their field and commercial release. On the other hand, CRISPR/Cas mutation analysis is extremely challenging because of the nature of α-gliadin genes which comprise approximately 100 copies of genes and pseudogenes disposed in tandem at the Gli-2 loci of chromosomes 6A, 6B, and 6D [19]. Recently, 47 α-gliadin genes were characterized for the Chinese Spring variety by Huo et al. [20], whereas 45 different α-gliadin variants were identified for the BobWhite variety using amplicon sequencing [21]. The repetitive DNA composition of these regions along with the duplication of genes and their tandem organization hinders the genomic sequence assembly and subsequent InDels analysis.

Sanger sequencing and next-generation sequencing (NGS) are both highly sensitive methods to characterize CRISPR/Cas targeted mutations. Diverse bioinformatic tools, such as TIDE, DSDecode, ICE, TIDER, and more recently DECODR have been developed to detect mutations from PCR amplicons sequenced through Sanger sequencing [22,23,24,25,26]. Nevertheless, Sanger sequencing of PCR amplicons is time-consuming, particularly when a substantial number of samples must be screened. NGS lacks this Sanger sequencing downside, being an alternative with a reliable sensitivity for amplicon analysis [27]. Many tools have been developed to screen CRISPR/Cas9-induced mutations using NGS data: CRISPR-GA [28], CRISPResso [29], AGESeq [30], BATCH-GE [31], and Cas-analyzer [32]. However, all these approaches are inefficient for numerous samples, large InDel detection, and multi-copy gene families. Hi-TOM application partially solves this problem as it is presented for multiple samples and target site analysis [33]. It was developed for hexaploid wheat InDels detection, yet this system established the A-subgenome as the template to determine the length of InDels in the B- and D-subgenomes [33]. CRISPR-DAV can also analyze many samples with Burrows-Wheeler Aligner (BWA) and Assembly Based ReAlignment (ABRA) for the alignment of mutant sequences to a reference genome or amplicon sequence [34]. Other examples of CRISPR mutation analysis tools are CRIS.py and CRISPRpic. These are python-based scripts that compare each sequence of the edited organism to a previously established reference amplicon sequence (named ref_seq for CRIS.py) [35,36]. Recently, a web-based software named GOANA also proved to be efficient for high-throughput analysis of InDels in large scale NGS datasets [37]. This tool can process treatment and control samples simultaneously and more than 1300 target sites in just over 20 min. However, in all the cases mentioned so far, a reference sequence needs to be established before InDel detection. This is not appropriate for the analysis of edited lines from a multi-copy gene family; the same reference sequence is not valid as each edited sequence needs to be compared to the closest reference sequence from the wild type (WT) amplicons.

In this work, we present a workflow to analyze InDels from the multi-copy α-gliadin gene family from wheat. It is based on NGS data without the need to previously establish a reference sequence for each mutated sequence and genetic background, a step that is needed in all InDel analysis tools revised in the literature. Moreover, the pipeline can handle different genotypes, target sites, and lines from different generations. The pipeline could be adapted to analyze InDels in other multigene families targeted with CRISPR/Cas. The workflow was tested in a multiple sample set, including three generations of edited wheat lines (T0, T1, and T2), from three different backgrounds and ploidy levels (hexaploid and tetraploid). Implementation of Bayesian optimization of Usearch [38] parameters, inhouse Python, and bash scripts are reported. All scripts are publicly available in a GitHub repository (https://github.com/LabFBARRO/CRISPRanalysis, accessed on 10 September 2021).

## 2. Results

### 2.1. Bayesian Optimization of Bioinformatic Pipeline Parameters in WT Lines

Before the optimization step, we constructed a non-redundant α-gliadin amplicon database using 35 sequences from the bread wheat RefSeq reference genome v1.1 [39], 213 sequences from the National Center for Biotechnology Information (NCBI) database, and 244 in-house α-gliadin sequences from wheat genotypes (Table S1) [19]. To that, primers expanding the α-gliadin amplicon were aligned to the complete α-gliadins sequence to extract the amplicon. Amplicons that had 100% of similarity were removed from the database to avoid over-representation of these sequences.

Bayesian optimization was implemented to optimize Usearch v9.2.64 parameters from merge to search steps for the α-gliadin amplicons on WT lines [38]. Briefly, the Usearch pipeline comprised: (i)—fastq_mergepairs for assembly of forward and reverse paired-end sequences; (ii)—fastq_filter for trimming low quality merged reads; (iii)—fastx_uniques for de-replicating filtered merged reads; (iv)—unoise2 for denoising de-replicated filtered merged reads, chimera detection, sequencing error corrections, and generating operational taxonomic units (OTUs) for wheat lines [40]; and (v)—search_global for the search of sequences in the OTUs database and the α-gliadin amplicon database to obtain the number of assigned reads to each one. OTUs are often treated as different “species” in taxonomic profiling applications [41], and this is not the case in the present work. Thus, we renamed OTUs as unique denoised amplicons (Amps) to avoid any misinterpretation of results. Among these steps, five parameters were included in the optimization protocol (Table 1). The optimized values were selected according to the total number of matched reads against the α-gliadin amplicon database; the highest value being the optimum. The optimal parameters values obtained are also listed in Table 1 and the Bayesian optimization convergence plot can be visualized in Figure S1. Results of the optimized pipeline for WT lines are in Table 2. This optimized workflow for WT lines retrieved 68.83% of raw reads after denoising and 95.08% of these were successfully matched to the α-gliadin database.

**Figure 1:**
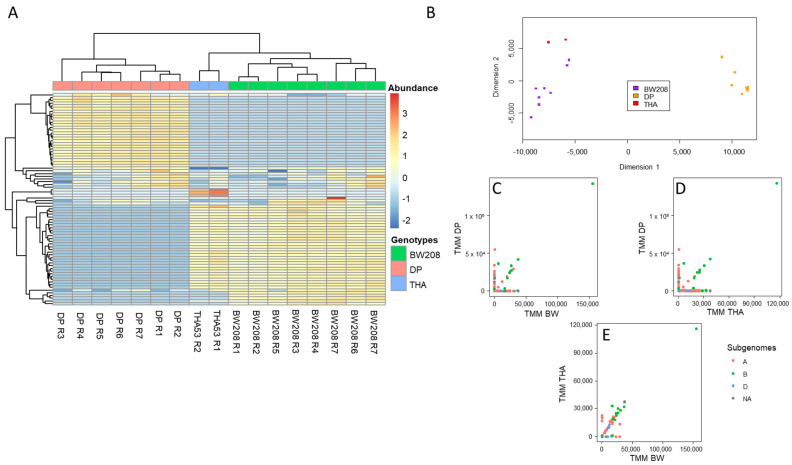
Abundance of α-gliadin Amps between wheat subgenomes A, B, and D in WT lines (BW208, THA53 and DP). (**A**). Heatmap of the abundance of Amps (rows) per WT line (columns). Dendrograms are represented for sequences (left) and WT lines (above). (**B**). Multidimensional scaling (MDS) plot of the Amp abundances for WT lines. (**C**). Comparison of Amp abundances between BW208 (hexaploid) and DP (tetraploid). (**D**). Comparison of Amp abundances between THA53 (hexaploid) and DP (tetraploid). (**E**). Comparison of Amp abundances between THA53 (hexaploid) and BW208 (hexaploid). WT: wild type. DP: Don Pedro. Amps: unique denoised amplicons. NA: Amps not assigned to a subgenome.

Total reads assigned to each Amp per sample were normalized by Trimmed Mean of M-values (TMM) for inter-sample comparisons of their abundance [42]. These abundances were represented for each WT line in a heatmap and MDS plot (Figure 1A,B). In these representations, lines were clustered by the similarity of their Amp abundance profiles. As shown in both graphs, lines were grouped according to their genetic background and ploidy level: hexaploid or tetraploid. It was possible to assign each Amp to either A, B, or D subgenomes by the presence of distinctive motifs in their translated peptide sequence as described in Van Herpen et al. [43]. Moreover, the two-by-two line comparison of Amp abundances clearly showed that Amps that belonged to the D-subgenome were absent in tetraploid lines (DP) (Figure 1C–E). In addition, the two-by-two line comparison also allowed the identification of Amps that are shared by the three genotypes (Figure 1C–E).

### 2.2. Implementation of Optimized Bioinformatic Pipeline in WT and CRISPR Lines

The pipeline was applied with optimized parameter values to obtain the normalized abundances (TMM-values) of Amps for all lines (WT and CRISPR), and to compare each CRISPR line with its corresponding WT line. As shown in Table 2, the optimized workflow retrieved 63.45% of all raw reads after denoising and 85.94% of these were matched to the α-gliadin database.

#### 2.2.1. InDel Identification and Characterization in CRISPR/Cas9 Lines

Based on the normalized abundances, we classified Amps with frequencies higher than 0.3% in two categories attending to their presence in WT and CRISPR lines. The first category was denoted as “WT Amps”: unique denoised amplicons present in the WT lines. The second one was named “CRISPR Amps”: unique denoised amplicons present in CRISPR lines and absent in WT lines, and therefore considered as new generation Amps containing putative targeted mutations.

**Figure 2:**
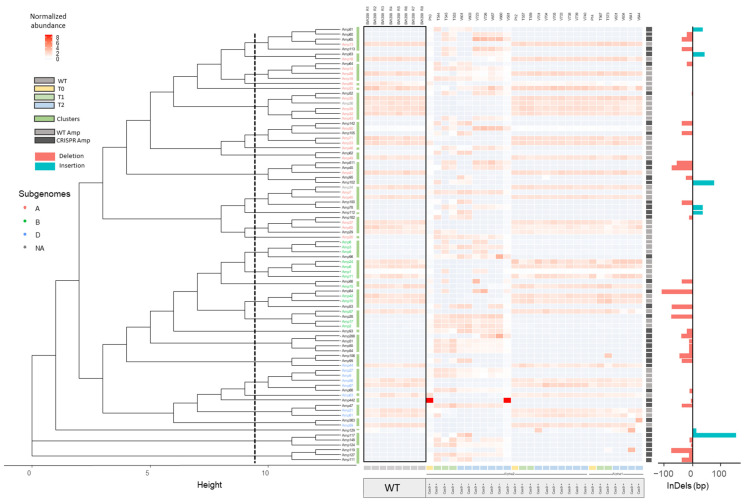
Dendrogram of Amps presented in lines with BW208 background, and their TMM normalized abundances represented as a min-max scaling heatmap. CRISPR Amps (only present in CRISPR lines) are represented in dark grey squares while WT Amps are represented as light grey squares. Clusters of Amps (green bars) were established according to the dendrogram (**left**) and were aligned through MUSCLE manually to detect the InDels size in CRISPR Amps (**right**). Relationship between lines (T0, T1, T2) and the absence or presence of Cas9 is also depicted per each line. Only Amps present in at least one line with a frequency higher than 0.3% per line were considered for the analysis. WT: wild type. Amps: unique denoised amplicons. NA: Amps not assigned to a subgenome.

**Figure 3:**
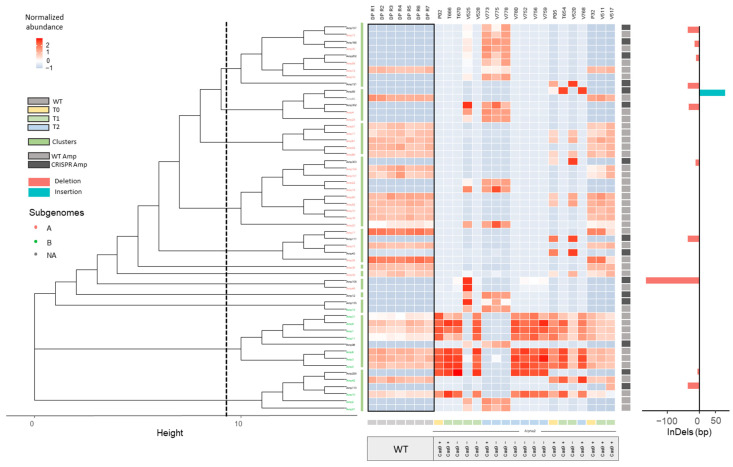
Dendrogram of Amps presented in lines with DP background, and their TMM normalized abundances represented as a min-max scaling heatmap. CRISPR Amps (only present in CRISPR lines) are represented in dark grey squares while WT Amps are represented as light grey squares. Clusters of Amps (green bars) were established according to the dendrogram (**left**) and were aligned through MUSCLE manually to detect the InDels size in CRISPR Amps (**right**). Relationship between lines (T0, T1, T2) and the absence or presence of Cas9 is also depicted per each line. Only Amps present in at least one line with a frequency higher than 0.3% per line were considered for the analysis. WT: wild type. Amps: unique denoised amplicons. NA: Amps not assigned to a subgenome.

**Figure 4:**
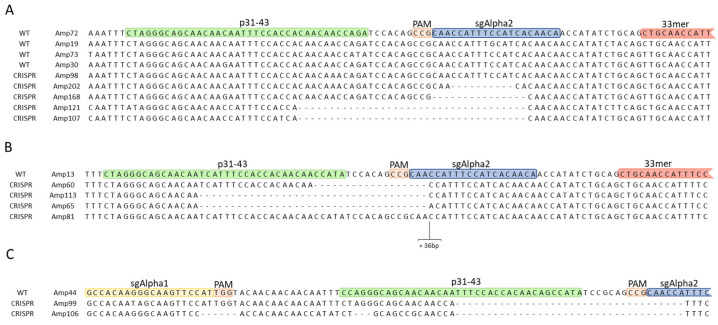
MUSCLE alignment of Amps clusters from the neighbor-joining dendrogram. (**A**) Cluster 1 of Amps present in lines with DP genotype. (**B**) Cluster 1 of Amps present in lines with BW208 genotype. (**C**) Cluster 22 of Amps present in lines with BW208 genotype. DP: Don Pedro. Amps: unique denoised amplicons.

After this classification, a dendrogram was constructed with all Amps for each background (BW208, DP, and THA53) based on sequence similarities. These dendrograms allowed us to group CRISPR Amps in clustersf along with their most closely related WT Amp. In addition, a heatmap was produced including normalized abundances of Amps per line and highlighting the presence of Cas9 coding sequence determined by PCR for each line. Then, we analyzed the results for each genetic background separately: hexaploid BW208 wheat (Figure 2), tetraploid DP wheat (Figure 3), and hexaploid THA53 wheat (Figure S2). To that, each Amp cluster determined by the dendrogram was aligned through different methods by Geneious software enabling InDel characterization in both CRISPR/Cas target sites: sgAlpha1 and sgAlpha2 (Figure 4). When a CRISPR Amp was grouped in a cluster not containing a WT Amp, the alignment and InDel characterization was performed with adjacent clusters. As shown in Figure 2 and Figure 3, deletions were the most common type of InDels in all cases, but insertions were also present in some lines.

For BW208 lines (Figure 2), we observed different profiles of Amp abundances between the WT and certain CRISPR T0 lines, which is highlighted in the heatmap. Moreover, analyzing the offspring for those lines, it is also shown that this difference was maintained in the next T1 and T2 generations. For example, the T544 line (a T1 descendant from the P10 line) had a similar profile of Amp abundances as its T2 descendants (V601 and V603 lines) but different from the WT lines. This behavior was also observed in the rest of the T1 descendants from the P10 T0 line (T545 and T553) and their T2 descendants. In contrast, the rest of the T0 lines (P12 and P14) presented a more likewise profile of Amp abundances to that of the WT lines. The usefulness of our approach is evident when we look at individual Amps. For example, Amp 442, present in the P10 T0 line and a T2 descendant (V581) with a high abundance (4413.08 and 4364.69 TMM, respectively), was absent in the remaining P10 descendants; the T1 parent to V581 line was not included in this study.

Comparable results were obtained for the tetraploid DP lines (Figure 3). In this case, the P02 and P05 T0 lines showed distinctive profiles of Amp abundances between them and in comparison to the WT lines and the P32 T0 line, which can be related to the different efficiency of CRISPR/Cas9. Once more, this dissimilarity was conserved between the T1 and T2 generations in most cases. Some T2 descendants from the T666 T1 line (V773, V775, and V778 lines) were an exception to this behavior, as they presented a different profile of Amp abundances as their T1 parent line. This could be explained by the presence of the CRISPR/Cas9 construct in the T1 line (T666). It is worth mentioning that 16 out of the 18 Amps which were not present in P02 and P05 progeny but present in WT lines, were assigned to sequences from the A subgenome.

In THA53 CRISPR lines (Figure S2), only the P20 T0 line and its descendants were found to have some differences in their profiles of Amp abundances compared to the WT lines, although they were not as evident as the rest of genetic backgrounds.

#### 2.2.2. Offspring Analysis in CRISPR/Cas9 Lines

The workflow described here is also of interest to analyze the CRISPR/Cas9 InDels through several generations and detect possible anomalies in their inheritability. A detailed analysis of the number of CRISPR Amps, and the number of putative targeted and non-targeted WT Amps for each T0 line and their T1 and T2 offspring are shown in Table 3, Table 4 and Table S2 for BW208, DP, and THA53 lines, respectively. As described in Section 2.2.1, the term ‘CRISPR Amps’ referred to unique denoised amplicons present in CRISPR lines but absent in WT lines, and therefore they were considered as new generation Amps probably as a consequence of CRISPR/Cas mutations. The number of Amps present in each line (with frequency > 0.3%) was named as ‘Total Amps/line’. The WT Amps that were found in CRISPR lines were considered as ‘Non-targeted WT Amps’, as they were not mutated by the CRISPR/Cas system. Finally, those WT Amps not detected in CRISPR lines were considered as ‘Putative targeted WT Amps’.

For the BW208 background (Table 3), the P12 and P14 T0 lines and their offspring presented a similar number of total Amps as the WT line. Moreover, CRISPR Amps were only detected in some of these T1 and T2 lines. Interestingly, for V701, V704, and T573 lines only one CRISPR Amp was found but more than one (2–3) putative targeted WT Amps were detected. The opposite was observed in the V641 line, which presented 2 CRISPR Amps but only one putative targeted WT Amp. In contrast, the P10 line and its descendants had a high number of CRISPR Amps while the number of non-targeted WT Amps was much lower than those of P12 and P14 lines. In addition, the number of putative targeted WT Amps in the P10 line and its offspring was also high (Table 3).

For the tetraploid DP background, CRISPR lines showed a wide range of variability for the number of putative targeted WT Amps (Table 4). The V773 (T2, Cas9 +) and V778 (T2, Cas9 −) lines showed the lowest number of non-targeted WT Amps. Despite this, only 4–5 CRISPR Amps were found in these lines (Table 4). Results for THA53 lines showed a lower number of CRISPR Amps than that of BW208 and DP (Table S2). It is outstanding that for some THA53 lines we found CRISPR Amps but the complete set of WT Amps (Table S2).

### 2.3. qPCR Amplicon Copy Number in CRISPR/Cas9 Lines

As mentioned above, we found that some lines and their descendants had a low number of CRISPR Amps but a high number of putative targeted WT Amps. This is particularly noteworthy for the tetraploid DP lines. For instance, the P02 T0 line presented 21 Amps in total—while WT lines presented 40—and only 2 out of 21 Amps were CRISPR Amps (Table 4). The P05 line exhibited a similar behavior (Table 4), with a total of 25 Amps detected but only 3 were CRISPR Amps. We performed a quantitative PCR (qPCR) to determine the copy number of the amplicons for these lines and compare it to that obtained for the WT to elucidate any possible genomic rearrangement after Cas9 cleavage.

We chose line V775 (T2, Cas9 –), which is derived from the P02 line, and for which we also had seeds in a T5 generation. The qPCR experiment confirmed that the V775 line and its T5 descendant presented lower copies of the amplicon than the WT, being the ratios for the amplicon copy number between the CRISPR and the WT lines of 0.24 and 0.21, for the V775 T2 and V775 T5 lines, respectively (Figure S3A). This result confirms that the number of α-gliadin amplicons present in these CRISPR lines is lower than in WT.

## 3. Discussion

Gluten proteins are composed of gliadins and glutenins and provide wheat dough with their unique viscoelastic and organoleptic properties [5]. However, they are also responsible for triggering the immune response in CD patients [7,8]. Among gluten proteins, the wheat α-gliadins contain the most immunogenic CD-epitopes [11]. Therefore, they are an attractive target for CRISPR/Cas to generate non-transgenic edited lines devoided of CD immunogenic epitopes, which could serve as a raw material for the production of foodstuffs that meet quality and safety expectations [17,44].

However, the characterization of the InDels produced by CRISPR/Cas is highly challenging in the α-gliadin family, which comprises approximately 100 copies of genes and pseudogenes disposed in tandem at *Gli-2* loci in chromosome 6 of each A, B, and D subgenomes [19]. Moreover, the exact copy number of these genes and their specific chromosome distribution are still uncertain. For Chinese Spring and BobWhite, 47 and 45 α-gliadin genes have been characterized, respectively [20,21]. This high variability found in WT lines along with the similarity of these sequences makes the analysis of InDels very complex.

Along with CRISPR/Cas edited lines, effective, accurate, and economic screening methods have been developed to characterize the InDels produced. NGS has increased in popularity over Sanger sequencing for the analysis of mutations caused by CRISPR/Cas because it allows studying the abundance of each unique mutation, works with many samples, and is less time-consuming. In all approaches based on Sanger sequencing and NGS sequencing [22,23,24,25,26,27,28,29,30,31,32,33,34,35,36,37], a reference gene is needed for mutation analysis. This is insufficient for the characterization of InDels from a multi-copy gene family as the α-gliadins from wheat. Therefore, a proper methodology is still needed to select the closest reference WT sequence for each newly generated CRISPR sequence to characterize InDels efficiently in the α-gliadins gene family and other multi-copy gene families.

An approximation proposed by Jouanin et al. uses droplet digital PCR (ddPCR) to identify small (1–50 bp) and large (>300 bp) InDels in wheat α-gliadin genes targeted with CRISPR/Cas [45]. However, the authors also stated the need to further perform deep sequencing to characterize these mutations. For this purpose, they developed GlutEnSeq; an in-solution gluten exome capture system [46]. In this approach, captured gluten sequence reads were mapped to the Chinese Spring reference genome. This could make difficult the characterization of sequence mutations, as plenty of polymorphisms have been observed in the α-gliadin genes between wheat varieties. Consequently, they proposed mapping reads from edited lines against a *de novo* assembly of reads from control lines.

In the present work, we have developed a quick workflow that enables the characterization and analysis of α-gliadins mutations induced by CRISPR/Cas9 using two sgRNAs in multiple lines and generations. We used NGS data from an α-gliadins amplicon which included two sgRNAs targeted regions [17]. The paired-end reads were then merged, filtered, de-replicated, denoised, and matched against a unique amplicon database using the Usearch algorithm [38]. Usearch is commonly used for microbial amplicon sequencing studies due to its high overall performance enriched by its Unoise pipeline [40,47]. VSEARCH is an open-source alternative that uses a heuristic method to identify similar sequences between the query and target ones in a similar manner as Usearch [48]. In our pipeline, both can be used for achieving the high-throughput InDel analysis of amplicons.

First, we implemented the Bayesian optimization of the α-gliadins amplicon analysis pipeline in WT lines. In our case, the objective function was the complete Usearch workflow from merge of raw reads to search sequences in the Amps (unique denoised amplicons) database and against a non-redundant α-gliadin database, previously constructed. The optimization of the pipeline was efficient, as it was able to retrieve Amps of biological importance; 95.08% of Amps matched the α-gliadin database, proving the efficiency of the pipeline in recovering α-gliadin Amps. The normalization was considered of essential relevance for inter-sample comparisons, and the TMM normalization method was used given its high performance [42,49]. Our workflow allowed the graphical representation of Amps abundances in a heatmap and MDS plot (Figure 1A,B). Notably, the samples clustered according to genotype and ploidy level, which confirms that many samples can be treated simultaneously regardless of their genetic background. Moreover, the approach allows the identification of Amps shared by the hexaploid genotypes and easily identifies those belonging to the D subgenome and not present in the durum wheat genotype (AABB).

After optimization, the bioinformatic pipeline was run including all lines; both CRISPR and WT lines. CRISPR Amps, present in CRISPR lines but absent in WT lines, and WT Amps were used as input for dendrogram construction by the pipeline. This allowed us to select the most appropriate WT reference sequence for each CRISPR Amp, and perform sequence alignments accordingly to characterize InDels (Figure 4). This step, absent in NGS amplicon analysis methods for single genes, is key for InDel analysis in multi-copy gene families targeted with CRISPR/Cas. Another key point when working with many samples, from different generations or containing mutations produced by different sgRNAs, is to quickly identify the samples containing putative mutations by their abundance. To that, heatmaps with normalized abundances of Amps per line were also created through our workflow and added to the existing dendrograms (Figure 2, Figure 3, and Figure S2). In addition to this, the workflow also produced tables with the most relevant information to facilitate the interpretation of the results; Total number of Amps, CRISPR Amps, non-targeted WT Amps, and putative targeted WT Amps per line (Table 3, Table 4, and Table S2). The information depicted in our dendrograms, heatmaps, and tables is characteristic of our approach as it was designed for the characterization of mutations in a multi-copy gene family. Thus, it is lacking in all tools revised in literature; designed for single-copy genes [22,23,24,25,26,27,28,29,30,31,32,33,34,35,36,37]. The combination of all (dendrogram, heatmaps, and tables), enabled us to determine the best CRISPR lines per background within a glance; lines containing InDels, and with a distinctive abundance Amp profile as that of the WT lines are easily noticeable. Furthermore, it allowed us to study the inheritance and segregation of the InDels produced throughout different generations (T0, T1, T2) in all three genotypes, and associate this information with the presence or absence of Cas9 coding sequence for each line.

Thanks to the bioinformatic pipeline, it is possible to highlight the distinctive profiles of Amps abundances in CRISPR lines that contain InDels and are therefore different from WT profiles. For example, in the BW208 hexaploid wheat, it was the P10 T0 line and its descendants the ones with the most distinctive profile of normalized abundances compared to its WT line. This line and its offspring presented a high number of CRISPR Amps and putative targeted WT Amps, indicating that InDels were inherited throughout generations and suggesting a high mutation rate of the α-gliadin WT Amps. For these lines, almost 80% of WT Amps were targeted. These results were consistent with the protein characterizations from Acid-PAGE gels and RP-HPLC analysis made in previous research [17]. In these lines, the number of CRISPR Amps varied slightly between generations in most cases; this could be due to the presence and activity of the Cas9 in the T0 and T1 descendants. This transgenerational activity of CRISPR/Cas was reported previously by Wang et al. in hexaploid wheat [50]. They applied multiplex gene editing targeting to *TaGW2*, *TaLpx-1*, and *TaMLO* genes in hexaploid wheat and found that non-modified sgRNA targets in early generation lines can be edited in the following generation. Zhang et al. also reported that *OsRR22* gene mutations in rice could be stably transmitted to subsequent generations [51]. Moreover, they also observed new mutations within the T1 offspring of one line, suggesting continuous modifications of WT alleles in Cas9 + lines.

The pipeline developed in this study also detected less noticeable Amp abundances profiles. In the case of the hexaploid BW208 genotype, the P12 and P14 T0 lines and their offspring presented a more similar profile of Amps abundances to that of the WT. Moreover, only some of these T1 and T2 lines presented CRISPR Amps. Due to the high similarity in the α-gliadin WT sequences, different WT sequences could have been mutated generating the same CRISPR Amp, this could explain why some lines presented one CRISPR Amp but more than one putative targeted WT Amps. The opposite was also observed; the V641 line presented more CRISPR Amps than putative targeted WT Amps. In this case, various copies of the same WT Amp could have been mutated resulting in different CRISPR Amps. This has also been described in other studies [50,52,53,54]. Yang et al. targeted different *Brassica napus* family genes with specifics CRISPR/Cas constructions and nonidentical InDels were observed in different alleles of the same gene [53]. Interestingly, the P14 and P12 T0 lines (Cas9 +) did not present CRISPR Amps nor putative targeted WT Amps but some of their offspring did. In these cases, Cas9 could have been less active and/or the InDels produced could have been present in their T0 lines but without reaching the 0.3% frequency needed to be considered a CRISPR Amp for our pipeline.

For the hexaploid wheat THA53 background, the differences in Amp abundances were also present but to a lesser extent than in the BW208 P10 lines. These results were also reflected in protein characterizations performed in previous research [17]. Some THA53 CRISPR lines presented CRISPR Amps but also the complete set of WT Amps, which could indicate that targeted mutations were still in heterozygosis.

In the case of the tetraploid DP background, we found many differences in the abundances profile of Amps in the P02 and P05 T0 lines, which were also inherited in the T1 and T2 lines. Most of the WT Amps from the A subgenome were not observed in the P02 and P05 CRISPR lines and their progeny. These results were consistent with the protein characterization carried out in previous research [17], which showed the loss of the α-gliadins encoded by genes in chromosome 6A [55]. For these lines, about 75% of WT Amps were targeted by the sgRNAs, but only 4 CRISPR Amps were found, prompting us that a rearrangement may have occurred within the *Gli-2* loci as a consequence of the NHEJ-mediated DNA reparation process after Cas9 cleavage. To test this hypothesis, we used qPCR to determine the amplicon copy number in the WT and the V775 CRISPR line. qPCR results confirmed the lower number of α-gliadin amplicons in this CRISPR line compared to the WT. These results reinforced the hypothesis that some genetic rearrangement occurred in these lines due to Cas9 cleavage. Because α-gliadin genes are in tandem disposition on chromosomes 6A and 6B [19], a large deletion is the most suitable explanation. As reported previously, the presence of spaced site-specific double-strand breaks (DSBs) could be associated with large deletions in rice by CRISPR/Cas9 [56]. The presence of large deletions in α-gliadin genes, as these are arranged in tandem, was also reported in wheat CRISPR lines [46]. However, the mechanism and the extent of such rearrangements need to be studied in more detail in future works. Therefore, the bioinformatic pipeline is not only efficient in identifying and characterizing InDels in edited lines but also in detecting putative deletions derived from Cas9 activity.

The case of the T670 line and its progeny also displayed the utility of this pipeline to study the mutations in Cas9 negative lines across consecutive generations. Both T1 and T2 descendant lines had the same number of non-targeted WT Amps and putative targeted WT Amps, suggesting that the T670 line was not segregating. As reported by Tang et al. [57], the identification of non-segregating Cas-free mutants is laborious and inefficient. They designed CRISPR/Cas9 vectors with a fluorescent tag (sGFP) to identify transgene-free mutants based on the absence of GFP fluorescence in later generations. They also reported that mutations were stably transmitted to the next generation without newly induced mutations. The pipeline reported here, which addresses amplicon abundances, is suitable for detecting these non-segregating lines and can be used in combination with other techniques once Cas-free lines are evidenced.

Nowadays, there is an increasing amount of studies that focus on approaches using multiplexed strategies for multi-locus editing with CRISPR/Cas9 [58]. Multiplexed strategies require the sequencing of different PCR amplicons. This analysis method was used by Zhang et al. to test the efficiency of a multiplex system by targeting 6 of the 14 PYL families of ABA receptor genes in a single transformation experiment and later tracing the generated mutations through T0, T1, T2, and T3 generations [59]. This bioinformatic workflow can be used to achieve an efficient and less time-consuming multiplex data analysis from transgenerational NGS data, as it could establish the proper WT reference sequence for each targeted region from a pool of PCR amplicons. In addition, some studies target whole gene families, for which our InDels analysis workflow could be useful as well; Li et al. targeted the entire k1C gene family in sorghum with a CRISPR/Cas9 construct [54]. They were able to edit at least 12 out of 20 k1C gene family members and characterize those InDels partially thanks to the selection of the proper WT reference sequence through the construction of multiple Neighbor-Joining trees. Our pipeline is fully automatized and ready to use for other gene families with minor adaptations.

## 4. Materials and Methods

### 4.1. Plant Material

A total of 72 wheat NGS samples were used in this study. They correspond to 55 edited lines and 3 WT lines from three different backgrounds and three consecutive generations (Table S3). For WT lines, a total of 8, 7, and 2 replicates were included for BW208, DP, and THA53 genotypes, respectively. The T0 edited lines were previously produced in Sánchez-León et al. [17] using immature scutella from three wheat lines—two hexaploids (cvs BW208 and THA53) and one tetraploid line (cv Don Pedro; DP)—as the target for biolistic delivery of plasmids carrying the CRISPR/Cas9 system along with specific sgRNAs targeting the α-gliadins: sgAlpha2 (GGTTGTGATGGAAATGGTTG) or sgAlpha1 (GCCACAAGAGCAAGTTCCAT). All T0 lines and their offspring were examined by PCR for the presence of the Cas9 gene, and their protein profile was determined by Acid-PAGE electrophoresis.

### 4.2. Next Generation Sequencing Data

Amplicon sequencing was carried out at Fundación Parque Científico de Madrid (Cantoblanco, Madrid) using a MiSeq system (https://www.illumina.com, accessed on 10 September 2021) under a 2 × 280 paired-end sequencing procedure as described in Sánchez-León et al. [17]. The primers used for the PCR amplification were the forward primer aGli900F1 (GTTAGAGTTCCAGTGCCACAA) and the reverse primer 33mer1R2 (GGTTGTTGTGGTTGCGRATA). All NGS data are available under the accession BioProject numbers PRJNA354904 and PRJNA782791.

### 4.3. Construction of a Non-Redundant α-Gliadin Amplicon Database

**Figure 5:**
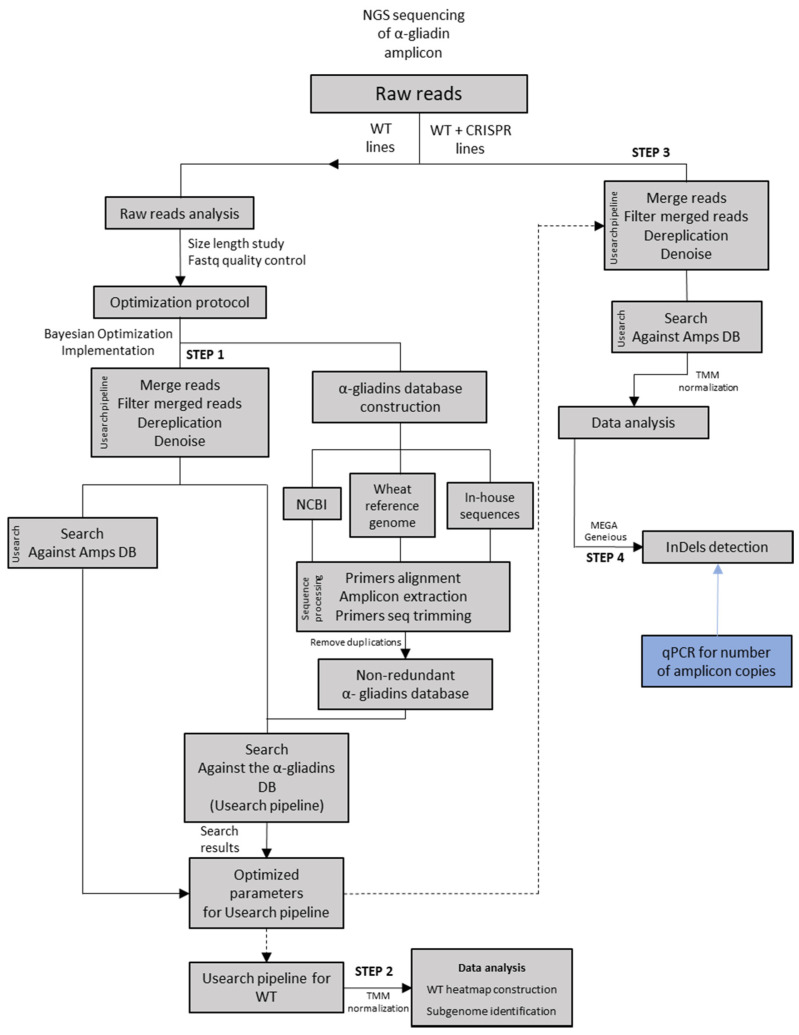
Pipeline for InDel analysis in edited amplicons of CRISPR lines, including prior optimization steps. Steps 1–4 can be found in CRISPR analysis repository: https://github.com/LabFBARRO/CRISPRanalysis, accessed on 10 September 2021. WT: wild type; Amps: unique denoised α-gliadin amplicons. Dashed lines indicate steps in which optimized parameters were used.

The α-gliadin database was created by using α-gliadin sequences from NCBI database (www.ncbi.nlm.nih.gov, accessed on 1 June 2021), the wheat reference genome [39], and in-house sequences from all three WT genotypes [19]. Amplicon primers were aligned on each database accession for amplicon extraction using the Geneious v2019.0.3 software (https://www.geneious.com, accessed on 10 September 2021), and both primers were trimmed from sequences. Duplicated sequences were removed to obtain a non-redundant database (Figure 5). The complete database for the α-gliadins is in Table S1.

### 4.4. Bayesian Optimization

Usearch v9.2.64 was used to process and characterize amplicon sequencing data [38]. This was run in a server with 64 cores and 128 GB of RAM with GNU/Linux Ubuntu version 18.04 (www.ubuntu.com, accessed on 10 September 2021, UK) as the operative system. To decide which values of Usearch parameters to apply in the pipeline, we implemented the Bayesian Optimization algorithm by Scikit-Optimize or skopt module from Python on WT lines (Figure 5) (Step 1 in CRISPR analysis repository: https://github.com/LabFBARRO/CRISPRanalysis). Skopt module is a simple and efficient library to minimize expensive and noisy black-box functions (https://github.com/scikit-optimize/scikit-optimize.git, accessed on 30 September 2021). In our case, the function is the Usearch pipeline that returned as last the total number of matched reads to the α-gliadins database. Briefly, the Usearch pipeline comprised merging raw reads, filtering low quality merged reads, de-replicating filtered merged reads, denoising de-replicated filtered merged reads, and the search of these sequences in the α-gliadins database. The optimization search minimized scores; thus, we returned the negative matched reads in the α-gliadin database to search for the maximum score. To establish the intervals of values for each parameter, we characterized the length of reads (Figure S4). The parameters for optimization were the following: diff and pct (merging step), maxee (filtering step), amp (denoising step), and id (search to database step).

To check the results of the Usearch pipeline optimization with WT lines, we normalized (TMM method) and analyzed the results of the pipeline with the optimized parameters (as in Section 4.5) (Step 2 in CRISPR analysis repository). The TMM normalization method estimates the scale factors between samples [42]. For TMM normalization, calcNormFactors function in edgeR package was used [60]. Optimized parameters were found for the pipeline comprising the following steps: (i) merging of forward and reverse reads for each line; (ii) filtering of high-quality merge reads, (iii) removal of duplicated filtered reads named as amplicon sequences, (iv) denoising of unique amplicon sequences, and (v) search of raw reads against (a) denoised unique amplicon sequences database or (b) against the α-gliadin database to optimize the process.

### 4.5. Optimized Protocol for CRISPR/Cas9 Edited Lines

Once we obtained the optimized parameters, we applied the pipeline for all wheat lines (WT and CRISPR lines) (Figure 5) (Step 3 in CRISPR analysis repository). For the last search step, in which reads are assigned to unique amplicon sequences, we attained the amplicon abundance raw data. To ensure that technical bias had minimal impact on final results, abundance data were then normalized using the TMM normalization method [42,49]. After normalization, the frequency of abundance was calculated for each unique amplicon and genotype. The unique denoised amplicons with frequencies lower than 0.3% were discarded for heatmap representation to minimize Illumina NGS errors [61,62].

### 4.6. Dendrogram Clusters and InDels Analysis

The unique denoised amplicon (Amp) abundances from WT and all lines obtained in Section 4.4 and Section 4.5, respectively, were analyzed with R [63]. This analysis consisted of the following steps: (i) application of a frequency threshold to Amp abundances, (ii) generation of the combination of dendrograms and heatmaps with the ggtree package, and (iii) generation of heatmaps for WT lines with the pheatmap package [64,65]. We implemented MEGA v10.1.7 software to establish the reference WT Amp for each CRISPR Amp (Amps present in CRISPR lines but absent in WT lines) according to the dendrogram obtained by the neighbor-joining method [66]. Subsequently, Geneious v2019.0.3 software (https://www.geneious.com) was used for dendrogram clusters alignment and InDel analysis using MUSCLE, MAFFT and ClustalW alignment methods [67,68,69]. Custom script with Python v3.6 [70] was also implemented to compare unique denoised amplicons between WT and CRISPR lines. The input for this script is the normalized Amp abundance table for all the lines while the output is a table with Amps presented in each line per genetic background (as Table 3, Table 4 and Table S2). The custom script enabled the visualization of data through generating tables containing the following: total Amps/line, CRISPR Amps, non-targeted WT Amps, and putative targeted WT Amps (Step 4 in CRISPR analysis repository) (Figure 5).

### 4.7. qPCR Analysis

Genomic DNA was isolated from leaf tissue following the hexadecyltrimethylammonium bromide (CTAB) method described in Stacey and Isaac, 1994 [71]. A qPCR was performed for the V775 and DP lines using SsoAdvanced Universal SYBR Green Supermix (Bio-Rad, USA) on CFX Connect Real-time PCR Detection System (Bio-Rad) (T_A_ = 62 °C). We used the DNA of the V775 T2 line and a T5 fixed-line descendent of the V775 line to confirm the results. The efficiency of each primer pair was determined by three serial dilutions (1:2) of a pool of both lines. The primers used for α-gliadin amplicon amplification were aGli900F1 and 33mer1R2 described previously in Section 4.2. The reference genes were the cell division control gene (*CDC*; Fwd, CAGCTGCTGACTGAGATGGA; Rev, ATGTCTGGCCTGTTGGTAGC) and the *Triticum aestivum* fatty acid desaturase 6 gene (*TaFad6*; Fwd, GCTTGGCATTCGGAAGGAGGAT; Rev, TCCGTCAGCTCAGCTTTGGCA), with 1 copy in durum wheat for haploid genome AB [72]. The *TaFad6* primers were designed for *TRITD6Bv1G038160* and *TRITD6Av1G024940* from *Triticum turgidum* reference genome Svevo.v1 [73].

The transformed efficiency for each target was calculated by the following formula:

$$E=\frac{E_{CFX}}{100}+1$$

Being *E_CFX_* the efficiency calculated by the CFX software.

Results for *E* for each target were: *E*_*TaFad*6_, 2.011; *E_CDC_*, 2.115; *E_α_*, 2.071.

The ratios between target and reference genes were calculated as:

$$Ratio=N\times \frac{{\left(E_{\left(ref\right)}\right)}^{MCq_{\left(ref\right)}}}{{\left(E_{\left(target\right)}\right)}^{MCq_{\left(target\right)}}}$$

Being *N* the number of copies per haploid genome for each reference gene (*TaFad6*, *N* = 2; *CDC*, *N* = 2), *E* the efficiency for reference and target genes, and *MCq* mean cycle quantification.

Both equations were adapted from Pfaffl et al. [74].

## 5. Conclusions

In the present study, we propose a workflow based on NGS amplicon data for the analysis of CRISPR/Cas targeted mutations in α-gliadin multi-copy gene family. The pipeline uses the closest WT reference sequence to identify InDels in CRISPR edited sequences. This straightforward pipeline enables the accurate identification and characterization of CRISPR/Cas edited lines by their InDels abundance, allowing to study the inheritability of mutations throughout different generations and genetic backgrounds. Moreover, it also detects putative large mutations in tandemly organized genes, such as the wheat α-gliadins, due to chromosomal rearrangements after Cas9 cleavage. This pipeline could be extended to the mutation analysis of other gene families targeted with CRISPR/Cas and used to select the most appropriate edited lines carrying the desired mutations.

## Figures and Tables

**Table 1:** Parameters, commands, and options used in the Usearch pipeline, as well as defined interval values and final optimal values selected throughout Bayesian optimization. Only WT Illumina MiSeq pairs from the α-gliadin amplicons were used to maximize the number of reads matched to the α-gliadins database. WT: wild type.

Parameter	Command	Option	Interval	Optimal Value
Merge	fastq_mergepairs	fastq_maxdiffs	(5–82)	58.94
Merge	fastq_mergepairs	fastq_maxdiffpct	(2–30)	24.66
Filter	fastq_filter	-	(0.25–1.5)	1.13
Dereplication	fastx_uniques	-	-	-
Denoising	unoise2	minampsize	(2–30)	22.47
Search	search_global/search_exact		0.99 and 1	0.99

**Table 2:** Percentage of reads which exceeded at each step of the optimized Usearch pipeline following the protocol with all WT lines raw dataset (left) and all lines, including WT and CRISPR lines, raw dataset (right).

	% of Reads	
	WT Lines	All Lines
Pairs (raw reads)	100 (8,205,317 reads)	100 (39,180,517 reads)
Merged	86.32	85.81
Filtered	88.57	87.47
Denoised	68.83	63.45
Matched to the amplicon database ^a^	95.08	85.94
Assigned to Amps ^b^	73.41	70.69

WT: wild type, Amps: unique denoised amplicons. ^a^ Percentage of reads contained in Amps matched to the α-gliadin database from NCBI, RefSeq reference genome v1.1, and in-house sequences. ^b^ Percentage of raw reads assigned to Amps.

**Table 3:** Total number of Amps (frequency > 0.3%) detected for each T0 line and their T1 and T2 offspring lines from the hexaploid BW208 genotype. We also represented the number of CRISPR Amps, non-targeted WT Amps, and putative targeted WT Amps per line. The number of Amps for the WT has been calculated as the mean of all WT lines.

T0	T1	T2	Cas9 +/−	Total Amps/Line ^a^	CRISPR Amps ^b^	Non-Targeted WT Amps ^c^	Putative Targeted WT Amps ^d^
WT			NA	48	NA	NA	NA
P10	-	-	Cas9 +	46	28	18	30
	T544	-	Cas9 +	32	20	12	36
		V601	Cas9 −	34	23	11	37
		V603	Cas9 −	34	23	11	37
	T545	-	Cas9 +	34	19	15	33
		V723	Cas9 +	33	18	15	33
		V726	Cas9 +	33	18	15	33
	T553	-	Cas9 +	37	24	13	35
		V657	Cas9 −	32	19	13	35
		V660	Cas9 −	33	21	12	36
	-	V581	Cas9 −	39	24	15	33
P12	-	-	Cas9 +	48	0	48	0
	T557	-	Cas9 +	48	0	48	0
		V701	Cas9 +	47	1	46	2
		V704	Cas9 −	47	1	46	2
		V705	Cas9 −	48	0	48	0
	T559	-	Cas9 +	48	0	48	0
		V733	Cas9 +	48	0	48	0
		V738	Cas9 +	48	0	48	0
		V739	Cas9 −	48	0	48	0
		V740	Cas9 −	48	0	48	0
P14	-	-	Cas9 +	48	0	48	0
	T567	-	Cas9 +	48	0	48	0
		V631	Cas9 +	48	0	48	0
		V634	Cas9 +	48	0	48	0
	T573	-	Cas9 +	46	1	45	3
		V641	Cas9 +	49	2	47	1
		V644	Cas9 +	48	1	47	1

T0: generation 0; T1: generation 1; T2: generation 2; WT: wild type; Cas +/−: presence or absence of Cas9 coding sequence; NA: not applicable; Amps: unique denoised amplicons. ^a^ Amps with frequencies > 0.3%. A more restrictive threshold was applied for considering putative targeted WT Amps in lines with no CRISPR Amps present. ^b^ Number of new generation Amps identified in CRISPR lines and absent in WT lines. ^c^ WT Amps detected in CRISPR lines. ^d^ Number of WT Amps not detected in CRISPR lines and considered as putative targeted Amps.

**Table 4:** Total number of Amps (frequency > 0.3%) detected for each T0 line and their T1 and T2 offspring lines from the tetraploid DP genotype. We also represented the number of CRISPR Amps, non-targeted WT Amps, and putative targeted WT Amps per line. The number of Amps for the WT has been calculated as the mean of all WT lines.

T0	T1	T2	Cas9 +/−	Total Amps/Line ^a^	CRISPR Amps ^b^	Non-Targeted WT Amps ^c^	Putative Targeted WT Amps ^d^
WT			NA	40	NA	NA	NA
P02	-	-	Cas9 +	21	2	19	21
	T666	-	Cas9 +	20	2	18	22
		V773	Cas9 +	14	4	10	30
		V775	Cas9 −	16	5	11	29
		V778	Cas9 −	15	5	10	30
		V780	Cas9 −	23	4	19	21
	T670	-	Cas9 −	16	2	14	26
		V752	Cas9 −	16	2	14	26
		V756	Cas9 −	17	3	14	26
		V759	Cas9 −	16	2	14	26
	V525	-	Cas9 −	20	8	12	28
	V528	-	Cas9 −	23	4	19	21
P05	-	-	Cas9 +	25	3	22	18
	T654	-	Cas9 +	17	1	16	24
		V768	Cas9 +	17	1	16	24
	V520	-	Cas9 −	28	4	24	16
P32	-	-	Cas9 +	40	0	40	0
	V511	-	Cas9 +	40	0	40	0
	V517	-	Cas9 +	40	1	39	1

T0: generation 0; T1: generation 1; T2: generation 2; WT: wild type; Cas +/−: presence or absence of Cas9 coding sequence; NA: not applicable; Amps: unique denoised amplicons; DP: Don Pedro. ^a^ Amps with frequencies > 0.3%. A more restrictive threshold was applied for considering putative targeted WT Amps in lines with no CRISPR Amps present. ^b^ Number of new generation Amps identified in CRISPR lines and absent in WT lines. ^c^ WT Amps detected in CRISPR lines. ^d^ Number of WT Amps not detected in CRISPR lines and considered as putative targeted Amps.

## Data Availability

Not applicable.

## Supplementary Materials

The following are available online at https://www.mdpi.com/article/10.3390/ijms222313076/s1.
