# Supplementary material for: A Bioinformatic Workflow for InDel Analysis in the Wheat Multi-Copy α-Gliadin Gene Family Engineered with CRISPR/Cas9
Source: Int J Mol Sci. 2021 Dec 3;22(23):13076. doi: 10.3390/ijms222313076 (PMC8657701; doi:10.3390/ijms222313076)
Supplement: Supplementary file 1 [file ijms-22-13076-s001.zip › Figure_S1_Convergence_fv.pptx]

## Slide 1
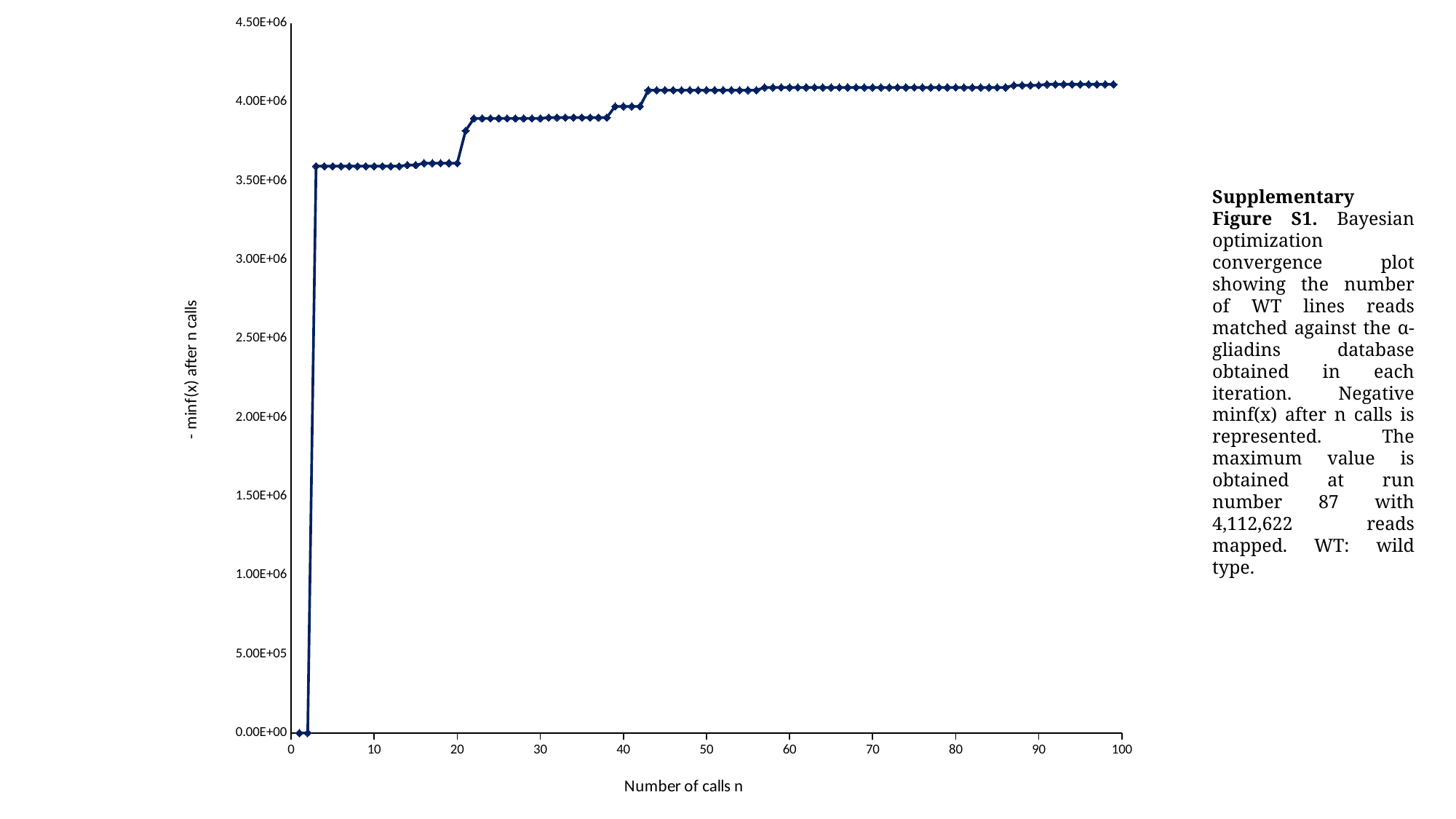

### Chart
| Category | - minf(x) after n calls |
|---|---|Supplementary Figure S1. Bayesian optimization convergence plot showing the number of WT lines reads matched against the α-gliadins database obtained in each iteration. Negative minf(x) after n calls is represented. The maximum value is obtained at run number 87 with 4,112,622 reads mapped. WT: wild type.
