# Supplementary material for: A Bioinformatic Workflow for InDel Analysis in the Wheat Multi-Copy α-Gliadin Gene Family Engineered with CRISPR/Cas9
Source: Int J Mol Sci. 2021 Dec 3;22(23):13076. doi: 10.3390/ijms222313076 (PMC8657701; doi:10.3390/ijms222313076)
Supplement: Supplementary file 1 [file ijms-22-13076-s001.zip › Figure_S2_Heatmap_THA_subgenomes_fv.pptx]

## Slide 1
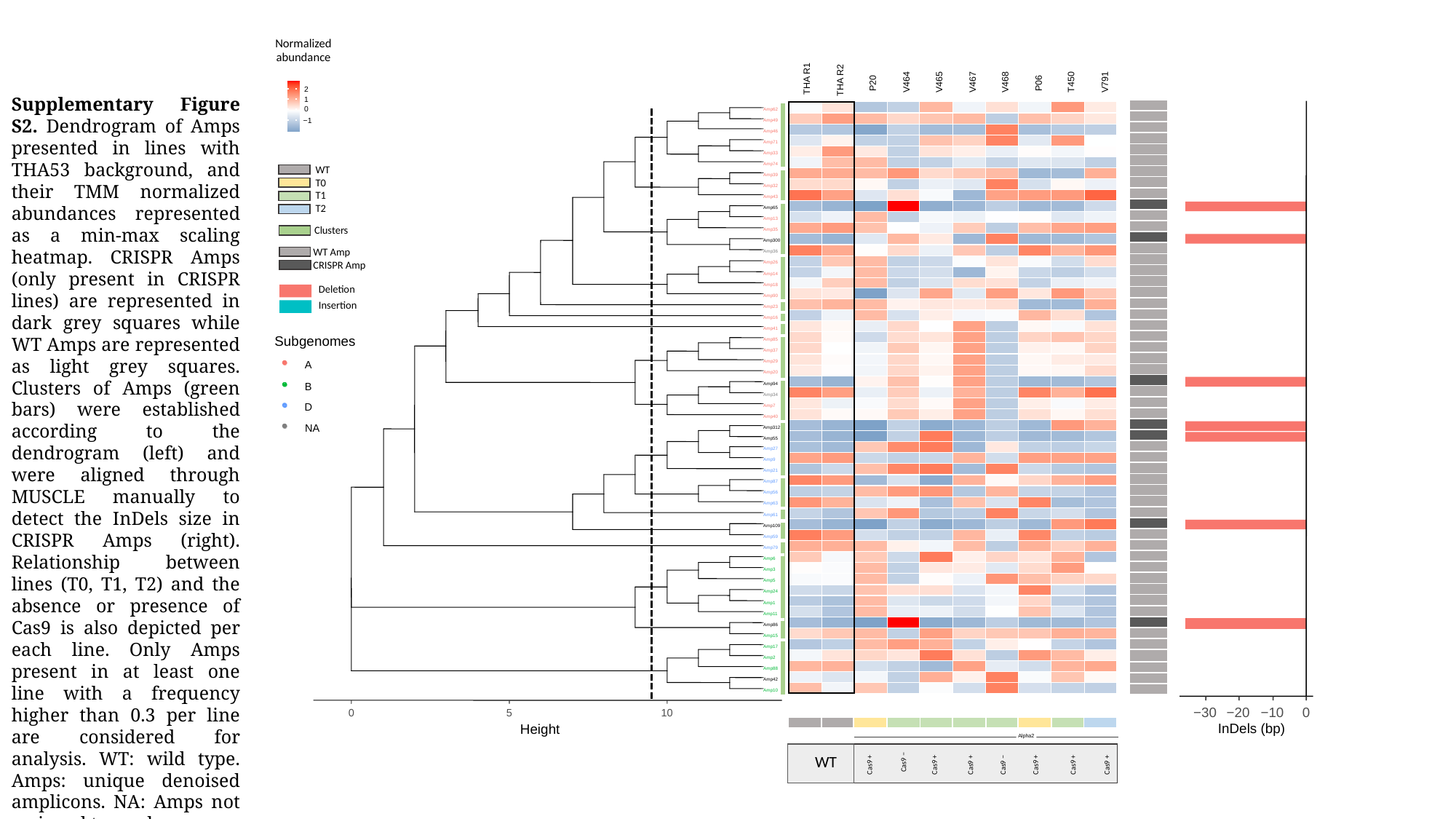

Normalized abundance
THA R1
THA R2
V464
V465
V467
V468
T450
V791
P20
P06
2
Supplementary Figure S2. Dendrogram of Amps presented in lines with THA53 background, and their TMM normalized abundances represented as a min-max scaling heatmap. CRISPR Amps (only present in CRISPR lines) are represented in dark grey squares while WT Amps are represented as light grey squares. Clusters of Amps (green bars) were established according to the dendrogram (left) and were aligned through MUSCLE manually to detect the InDels size in CRISPR Amps (right). Relationship between lines (T0, T1, T2) and the absence or presence of Cas9 is also depicted per each line. Only Amps present in at least one line with a frequency higher than 0.3 per line are considered for analysis. WT: wild type. Amps: unique denoised amplicons. NA: Amps not assigned to a subgenome.
1
−30
−20
−10
0
InDels (bp)
0
Amp62
−1
Amp49
Amp46
Amp71
Amp33
WT
T0
T1
T2
Clusters
WT Amp
CRISPR Amp
Deletion
Insertion
Amp74
Amp39
Amp32
Amp43
Amp65
Amp13
Amp35
Amp300
Amp36
Amp26
Amp14
Amp18
Amp90
Amp23
Amp16
Subgenomes
A
B
D
NA
Amp41
Amp85
Amp37
Amp29
Amp20
Amp94
Amp34
Amp7
Amp40
Amp312
Amp55
Amp27
Amp9
Amp21
Amp87
Amp56
Amp63
Amp61
Amp109
Amp59
Amp79
Amp6
Amp3
Amp5
Amp24
Amp1
Amp11
Amp86
Amp15
Amp17
Amp2
Amp88
Amp42
Amp10
0
5
10
Height
Alpha2
Cas9 −
Cas9 +
Cas9 +
Cas9 +
Cas9 +
Cas9 +
Cas9 +
Cas9 −
WT
