# Supplementary material for: A Bioinformatic Workflow for InDel Analysis in the Wheat Multi-Copy α-Gliadin Gene Family Engineered with CRISPR/Cas9
Source: Int J Mol Sci. 2021 Dec 3;22(23):13076. doi: 10.3390/ijms222313076 (PMC8657701; doi:10.3390/ijms222313076)
Supplement: Supplementary file 1 [file ijms-22-13076-s001.zip › Figure_S3_deletions_fv.pptx]

## Slide 1
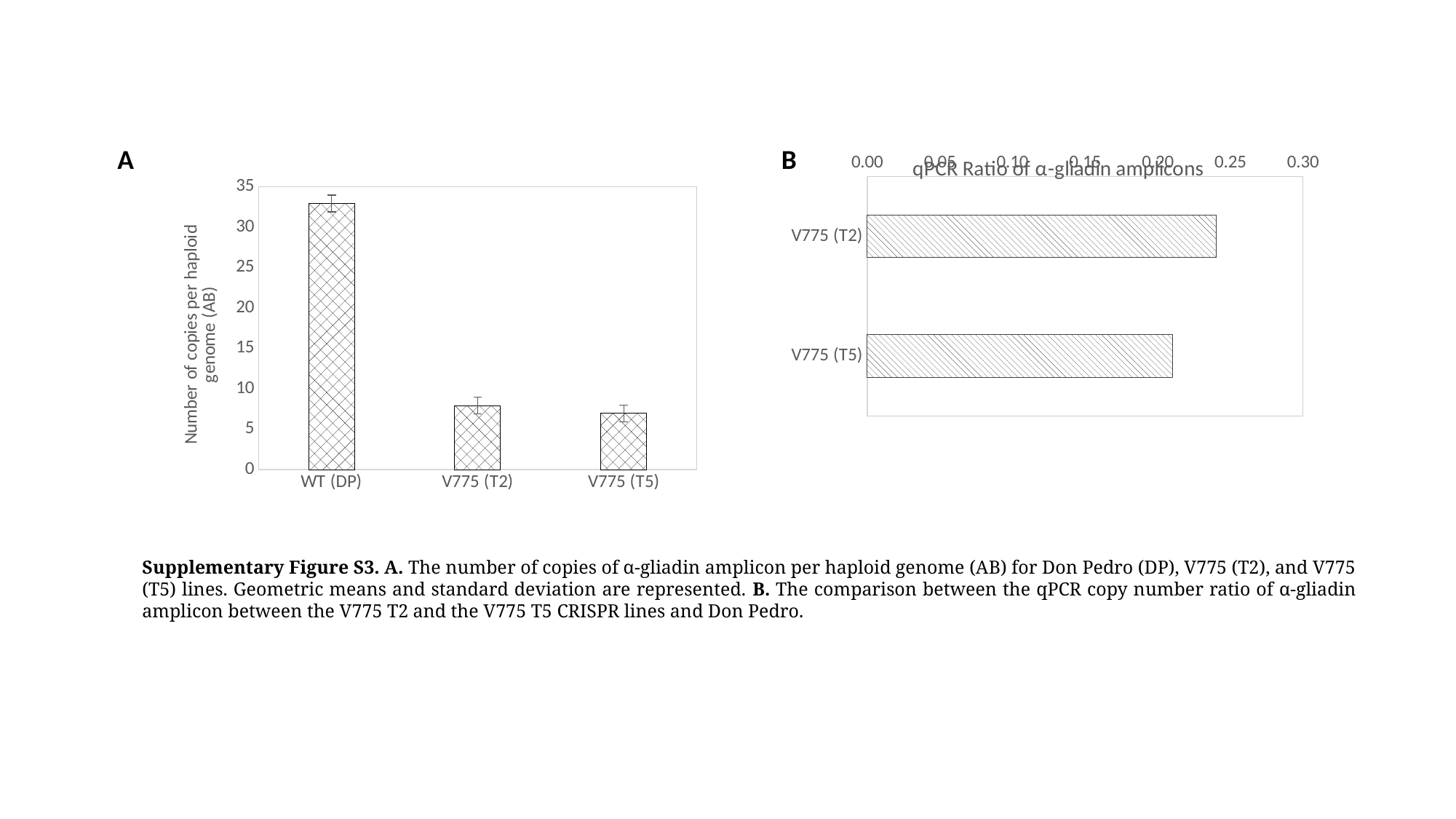

A
B
### Chart
| Category | DP vs B775 (T2) |
|---|---|
| V775 (T2) | 0.24032675924573738 |
| V775 (T5) | 0.21 |
### Chart
| Category | Mean |
|---|---|
| WT (DP) | 32.94 |
| V775 (T2) | 7.92 |
| V775 (T5) | 6.94 |Supplementary Figure S3. A. The number of copies of α-gliadin amplicon per haploid genome (AB) for Don Pedro (DP), V775 (T2), and V775 (T5) lines. Geometric means and standard deviation are represented. B. The comparison between the qPCR copy number ratio of α-gliadin amplicon between the V775 T2 and the V775 T5 CRISPR lines and Don Pedro.
