# Supplementary material for: A Bioinformatic Workflow for InDel Analysis in the Wheat Multi-Copy α-Gliadin Gene Family Engineered with CRISPR/Cas9
Source: Int J Mol Sci. 2021 Dec 3;22(23):13076. doi: 10.3390/ijms222313076 (PMC8657701; doi:10.3390/ijms222313076)
Supplement: Supplementary file 1 [file ijms-22-13076-s001.zip › Figure_S4_Length_amplicon_controls_fv.pptx]

## Slide 1
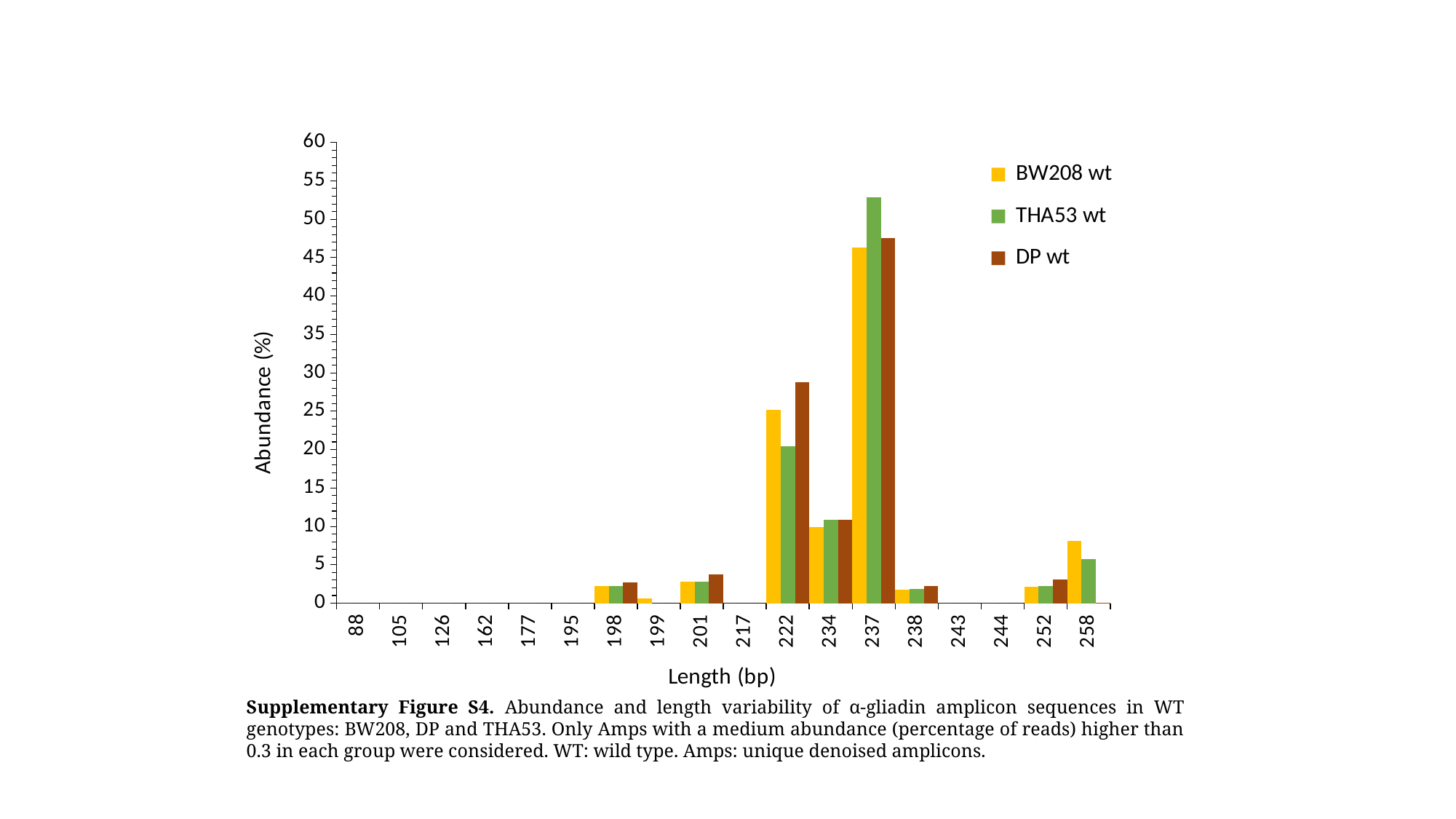

### Chart
| Category | BW208 wt | THA53 wt | DP wt |
|---|---|---|---|
| 88 | 0.0 | 0.0 | 0.0 |
| 105 | 0.0 | 0.0 | 0.0 |
| 126 | 0.0 | 0.0 | 0.0 |
| 162 | 0.0 | 0.0 | 0.0 |
| 177 | 0.0 | 0.0 | 0.0 |
| 195 | 0.0 | 0.0 | 0.0 |
| 198 | 2.2151549880744548 | 2.2377371353334405 | 2.6639849934349016 |
| 199 | 0.6163331406845187 | 0.0 | 0.0 |
| 201 | 2.7861114597590926 | 2.831035169114983 | 3.7702401603291715 |
| 217 | 0.0 | 0.0 | 0.0 |
| 222 | 25.194780350829905 | 20.438605151863865 | 28.760878313227195 |
| 234 | 9.914754478437208 | 10.900443570409113 | 10.833331274390499 |
| 237 | 46.346248023986305 | 52.88497763119333 | 47.5919923705587 |
| 238 | 1.783499807664723 | 1.8222310643976636 | 2.2424336289564177 |
| 243 | 0.0 | 0.0 | 0.0 |
| 244 | 0.0 | 0.0 | 0.0 |
| 252 | 2.0947065514710785 | 2.241171959168902 | 3.0696687448119673 |
| 258 | 8.071436755851481 | 5.745489202614222 | 0.009835063364586734 |Supplementary Figure S4. Abundance and length variability of α-gliadin amplicon sequences in WT genotypes: BW208, DP and THA53. Only Amps with a medium abundance (percentage of reads) higher than 0.3 in each group were considered. WT: wild type. Amps: unique denoised amplicons.
