# Supplementary material for: A Bioinformatic Workflow for InDel Analysis in the Wheat Multi-Copy α-Gliadin Gene Family Engineered with CRISPR/Cas9
Source: Int J Mol Sci. 2021 Dec 3;22(23):13076. doi: 10.3390/ijms222313076 (PMC8657701; doi:10.3390/ijms222313076)
Supplement: Supplementary file 1 [file ijms-22-13076-s001.zip › Table S2_THA table_fv.pdf]

Supplementary Table S2. Total number of Amps (frequency > 0.3%) detected for each T0 line and their T1 and T2 offspring lines from the hexaploid THA53 genotype. We also represented the number of CRISPR Amps, non-targeted WT Amps, and putative targeted WT Amps per line. The number of Amps for the WT has been calculated as the mean of all WT lines.

T0	T1	T2	Cas9 +/−	Total Amps/line[a]	CRISPR Amps[b]	Non-targeted WT Amps[c]	Putative targeted WT Amps[d]
WT			NA	47	NA	NA	NA
P06	-	-	Cas9 +	47	0	47	0
	T450	-	Cas9 +	49	2	47	0
		V791	Cas9 +	49	2	47	0
P20	-	-	Cas9 +	47	2	45	2
	V464	-	Cas9 −	50	3	47	0
	V465	-	Cas9 +	50	3	47	0
	V467	-	Cas9 +	46	1	45	2
	V468	-	Cas9 −	37	1	36	11

T0: generation 0; T1: generation 1; T2: generation 2; WT: wild type; Cas +/−: presence or absence of Cas9 coding sequence; NA: not applicable; Amps: unique denoised amplicons.

[a] Amps with frequencies > 0.3%. A more restrictive threshold was applied for considering putative targeted WT Amps in lines with no CRISPR Amps present.

[b] Number of new generation Amps present in CRISPR lines and absent in WT lines.

[c] WT Amps detected in CRISPR lines.

[d] Number of WT Amps not detected in CRISPR lines and considered as putative targeted Amps.
